# A Serious Digital Game (SugarVita) to Support Diabetes Self-Management: Pilot Randomized Controlled Trial

**DOI:** 10.2196/99345

**Published:** 2026-08-07

**Authors:** Edouard Reinders, Elma Cruts - Van de Meulengraaf, Ryan De Vries, Natal van Riel, Pieter Van Gorp, Jeanne Dieleman, Pleun Wouters - van Poppel, Harm Haak

**Affiliations:** 1 Internal Medicine, Maastricht University Medical Centre, Maastricht, Limburg, The Netherlands; 2 Care and Public Health Research Institute, Maastricht, Limburg, The Netherlands; 3 Department of Biomedical Engineering, Eindhoven University of Technology, Het Eeuwsel 53, Eindhoven, North Brabant, 5612, The Netherlands, 31 40 247 9111; 4 Internal Medicine, Máxima Medisch Centrum, Veldhoven, North Brabant, The Netherlands; 5 Science Office, Máxima Medical Center, Veldhoven, The Netherlands

**Keywords:** diabetes self-management and education support, digital education, serious games, eHealth, mobile app

## Abstract

**Background:**

Serious digital games have been proposed as a novel approach to support diabetes education and self-management, but evidence regarding their effectiveness remains limited.

**Objective:**

This study aimed to evaluate the effects of SugarVita, a serious game for people with type 2 diabetes, on diabetes-related knowledge, self-confidence, and self-management. Secondary outcomes included hemoglobin A_1c_ (HbA_1c_), engagement, and user evaluation.

**Methods:**

In this pilot randomized controlled trial, 30 adults with type 2 diabetes were randomized to SugarVita plus standard care or standard care alone for 8 weeks. Outcomes were assessed before and after the intervention using validated questionnaires and laboratory HbA_1c_ values. Within-group changes were analyzed using Wilcoxon signed-rank tests and between-group differences using Mann-Whitney *U* tests. Bonferroni correction was applied for multiple primary outcomes.

**Results:**

No statistically significant between-group differences were observed for diabetes-related knowledge, self-confidence, or self-management after correction for multiple testing. Both groups showed numerical improvements over time. HbA_1c_ decreased significantly within the intervention group (median 73.0, IQR 70.8-81.5 to median 64.5, IQR 60.8-72.0 mmol/mol; *P*=.007), whereas no significant change was observed in the control group. Greater total playtime was moderately associated with HbA_1c_ reduction. User evaluations indicated high perceived educational value.

**Conclusions:**

Participants reported positive experiences with SugarVita and perceived the game as educational and user-friendly. No statistically significant between-group differences were observed for the primary outcomes. These findings support the feasibility and acceptability of SugarVita as a digital educational intervention and warrant further evaluation in larger studies.

## Introduction

The global incidence of diabetes is on the rise, with projections estimating that nearly 580 million people worldwide will be affected by 2030 [1]. Traditionally, type 2 diabetes mellitus (T2DM) has been considered a disease that develops later in life. However, an increasing number of younger individuals are now being diagnosed [2]. Diabetes is associated with early and late complications that, despite advances in cardiovascular risk management, continue to drive an elevated risk of cardiovascular events [3]. Consequently, individuals with T2DM have higher mortality rates and a shorter life expectancy than those without the disease [4].

Effective disease management necessitates that patients understand the factors influencing blood glucose levels, such as diet, physical activity, and mental well-being [5]. Lifestyle changes, including healthier choices in daily routines, are often essential to achieve good regulation [6]. Better disease control results in fewer day-to-day complaints, a reduced risk of long-term complications, and improved life expectancy [7]. Therefore, early awareness of the disease, its contributing factors, and its consequences is crucial. Guidance from health care professionals, including general practitioners, internists, and specialized nurses, is vital in supporting this process. Traditionally, patient education has involved one-on-one sessions and ongoing counseling provided by these professionals.

Given the increasing prevalence of diabetes, the growing demand for health care professionals, and the associated medical costs, innovative solutions are required to ensure sustainable care. Current diabetes guidelines emphasize the use of digital tools for patient education [8]. The advantage of these tools, such as smartphones, lies in their widespread availability and easy access to diabetes-related apps through platforms such as the Google Play Store and the iOS Store. With the global proliferation of smartphones, these tools offer a promising means of providing diabetes education.

One innovative approach involves using educational games to teach patients about their disease in an engaging manner. These games aim to combine entertainment with education, with the intention of enhancing engagement and supporting learning compared to traditional one-on-one sessions. In recent years, there has been a surge in the development of digital diabetes games, as evidenced by their growing presence in both scientific literature and app stores [9]. SugarVita, a digital educational game, was developed from this perspective [10,11]. It aims to assist individuals with T2DM in managing their condition more effectively, strengthening their confidence in daily diabetes care, and improving their understanding of the disease.

Although interest in digital diabetes games has increased in recent years, evidence regarding their effectiveness remains limited. Previous studies have often focused on feasibility, usability, or user satisfaction, while effects on diabetes-related knowledge, self-management, and clinical outcomes have been evaluated less frequently. Therefore, additional research is needed to better understand the role of serious games in supporting diabetes education and self-management in daily practice [9].

The primary objective of this study was to evaluate the impact of playing SugarVita on diabetes-related knowledge, self-management, and self-confidence in individuals with T2DM, as these outcomes were considered the most direct measures of the educational objectives of the intervention. Hemoglobin A_1c_ (HbA_1c_) was included as a secondary outcome to explore whether improvements in diabetes education and self-management might translate into changes in glycemic control. Additional secondary objectives included analyzing player engagement data (eg, total playtime) and participant evaluations of the game (eg, perceived enjoyment and educational value).

## Methods

### Study Design and Participants

An 8-week randomized controlled trial was conducted between June 2024 and December 2024 at the Department of Internal Medicine, Máxima Medical Center, Eindhoven and Veldhoven, the Netherlands. The study was designed as a pilot randomized controlled trial including 30 participants to explore its impact on clinical outcomes and user experience.

All participants were recruited from the outpatient clinic of the Máxima Medical Center. Inclusion criteria were as follows: (1) a diagnosis of T2DM, (2) HbA_1c_ levels >64 mmol/mol at baseline, (3) BMI >25 kg/m^2^, and (4) aged ≥18 years. Exclusion criteria included: (1) pregnancy or breastfeeding, (2) ongoing treatment for malignancy, and (3) inability to speak Dutch.

During the first visit, participants signed informed consent forms and were randomized to either the intervention or control group. Randomization was stratified by age and baseline HbA_1c_ values. Participants were randomized using block randomization with sealed allocation lots prepared before the start of participant enrollment. Eligible participants were assigned to the intervention or control group only after completion of baseline procedures. Allocation was concealed until the moment of assignment. A brief overview of their medical history and current medication use was registered. In addition, changes in glucose-lowering medication occurring shortly before or during the study period were recorded and reviewed during follow-up. Additionally, HbA_1c_ levels from the participants’ most recent routine clinical visits were documented. All participants completed 3 questionnaires at baseline and again at the end of the 8-week study period, assessing diabetes self-confidence, self-management, and diabetes-related knowledge. The same questionnaires were administered at baseline and follow-up. As participants did not receive feedback on their responses, any practice effects due to repeated testing were expected to be limited and equally applicable to both study groups.

Participants in the control group did not play SugarVita and received standard care according to their individual treatment plans during the 8-week follow-up. Participants in the intervention group were instructed to download and play SugarVita on their own smartphone, tablet, or PC from the Google Play Store (Google LLC) or the Apple App Store (Apple Inc). Tablets (Samsung Galaxy Tab A6, Samsung Electronics) were provided by the research team if needed. After 1 week, participants of the intervention group received a follow-up phone call to address any technical issues related to logging into or using SugarVita. No additional educational or behavioral support was provided. At the start of the study, participants in the intervention group also received a paper-based assignment card to be completed over the 8-week study period. These challenges were designed to enhance engagement and motivate participants to continue playing the game throughout the study period. For example, in the fourth week, participants were asked to play the game with a relative or friend in a multiplayer setting (refer to Multimedia Appendix 1 for the assignment card).

During the second visit, at the end of the study, all participants completed the same 3 questionnaires again. Additionally, participants in the intervention group filled out an extra questionnaire evaluating their experience with SugarVita (Multimedia Appendix 2).

### Standard Diabetes Care

All participants received standard diabetes care in accordance with Dutch national guidelines for T2DM management, as outlined in the NHG-Standaard Diabetes mellitus type 2 [12]. Standard care consisted of regular outpatient follow-up with an internist and/or diabetes nurse specialist. During these consultations, lifestyle-related topics such as dietary habits, physical activity, weight management, medication adherence, and self-monitoring of blood glucose were routinely discussed.

Diabetes education was primarily delivered through individual consultations rather than structured group-based education programs. Participants also had access to written educational materials provided by the hospital or national diabetes organizations, covering general aspects of diabetes management and lifestyle modification.

No structured digital educational tools, mobile health apps, or serious games were part of routine care during the study period. Both the intervention and control groups received the same standard care. No additional educational sessions were introduced as part of the study protocol, apart from access to the SugarVita serious game for participants randomized to the intervention group.

### Ethical Considerations

The study was registered at ClinicalTrials.gov (NCT06392178) and conducted in accordance with the Declaration of Helsinki. The Medical Research Ethics Committee of Máxima Medical Center reviewed the study and determined that it does not fall under the scope of the Dutch Medical Research Involving Human Subjects Act (N24.020); therefore, formal ethics approval was not required. Eligible participants provided written informed consent prior to participation.

### Outcome Measures

#### Self-Confidence

Self-confidence in diabetes self-care was assessed using the validated Dutch questionnaire “Vertrouwen in Diabetes Zelfzorg” [13]. This instrument consists of 21 questions in which participants rate their confidence in performing various self-care tasks. Scores range from 21 to 105, with higher scores indicating greater self-confidence (Multimedia Appendix 3).

#### Self-Management

Self-management was assessed using the validated Dutch version of the Diabetes Management Self-Efficacy Scale [14]. This questionnaire consists of 20 items in which participants estimate their perceived ability to perform various tasks related to diabetes management. Total scores range from 20 to 100, with higher scores indicating greater perceived self-management capacity (Multimedia Appendix 3).

#### Diabetes-Related Knowledge

Diabetes-related knowledge was assessed using a translated version of the Diabetes Knowledge Test [15]. As no validated Dutch instrument was available at the time of the study, the original English version was translated into Dutch. The resulting questionnaire consists of 19 items related to diabetes knowledge. Total scores range from 0 to 59, with a maximum possible score of 59. The Dutch version used in this study has not yet been validated (Multimedia Appendix 3).

#### Hemoglobin A_1c_

HbA_1c_ values were obtained from routine clinical measurements documented in the electronic health record. No additional blood sampling was required. Given the duration of the intervention (8 weeks) and the expected delay before HbA_1c_ reflects sustained glycemic changes, postintervention values were included if measured within 12 weeks after study completion. Baseline HbA_1c_ values were accepted if collected within 12 weeks prior to study enrollment.

#### Player Data

Player activity was monitored remotely throughout the intervention period. Each participant was assigned an anonymous user account to access SugarVita. This enabled the research team to track detailed gameplay data, including login frequency, number of game sessions, session completion rates, total playtime, and in-game behavior. Gameplay data also included the number and accuracy of diabetes-related multiple-choice questions triggered during gameplay, the gameplay mode (solo vs multiplayer), and device type. Gameplay data were linked to study identifiers rather than personal information, stored securely, and accessible only to members of the study team.

#### Evaluation of SugarVita

Upon completion of the intervention, participants in the intervention group were invited to fill out an additional evaluation questionnaire regarding their experience with SugarVita. The questionnaire consisted of 8 items rated on a 10-point Likert scale (1=strongly disagree and 10=strongly agree), assessing aspects such as perceived educational value and level of challenge. Participants were also given the opportunity to provide open-ended comments to elaborate their responses.

### SugarVita

SugarVita was developed at our outpatient clinic in collaboration with Eindhoven University of Technology and in consultation with a group of patients with diabetes who volunteered for this project and did not participate in the current study. Various game types, including quiz and puzzle games, were tested, but patients preferred a digital version of a classic board game. The development process and selection criteria are described in a previously published article [11]. The core of SugarVita is based on the Eindhoven Diabetes Education Simulator, a physiology-based mathematical model that predicts blood glucose responses in a semipersonalized manner over 24 hours. The simulator incorporates key factors influencing glycemic control, allowing the game to simulate realistic glucose dynamics in response to player decisions [16].

**Figure 1.**
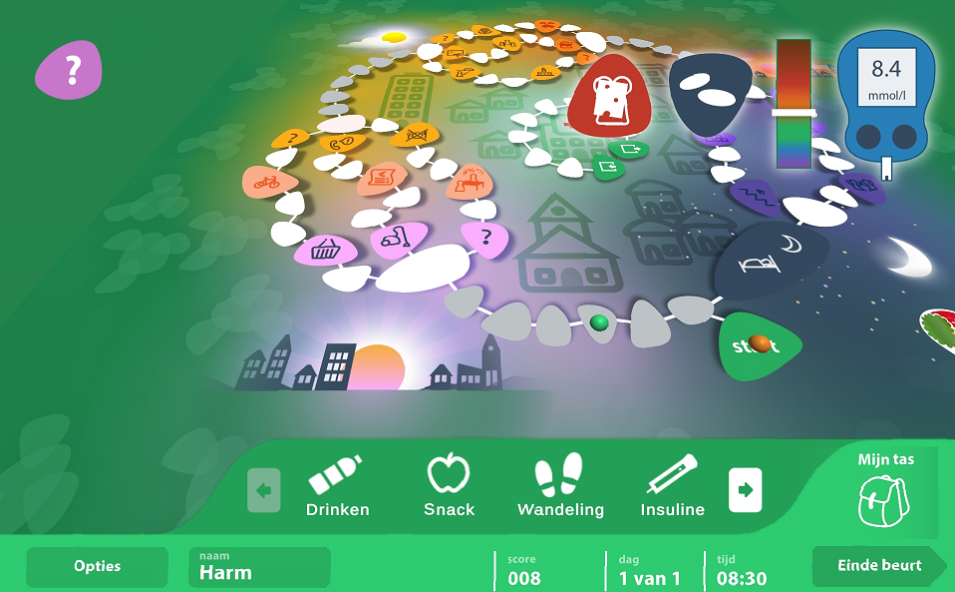
Screenshot of SugarVita.

SugarVita resembles the classic *Game of the Goose* or *Snakes and Ladders* game, with a board consisting of tiles and players rolling a digital die to advance. SugarVita can be played alone, with friends or family in multiplayer mode, or against a computer opponent. The game starts in the morning with medication intake and breakfast, then progresses through the day, with each tile representing 20 minutes of the day. When landing on a tile, players make choices regarding everyday events (eg, exercising, eating, snacking, and commuting). The player’s blood glucose level is continuously visible during the game and fluctuates based on their actions. Players earn the most points by maintaining their blood glucose levels within a certain range. Additionally, some tiles present random multiple-choice knowledge questions about diabetes, with correct answers earning extra points. The player with the most points at the end of the day wins the game. At the end of the virtual day, players receive a summary of their performance, including their glucose curve. Animated pawns (eg, a piece of fruit) are used to navigate the board, and completing certain challenges unlocks new pawns (refer to Figure 1 for an illustrative screenshot).

### Statistical Analysis

Within-group changes from baseline to follow-up were assessed using Wilcoxon signed-rank tests. Between-group differences in outcome scores (before and after) were evaluated using the Mann-Whitney *U* test. Given the evaluation of 3 primary outcomes, a Bonferroni correction was applied to diabetes-related knowledge, self-confidence, and self-management, resulting in a significance threshold of *P*<.017. Results are reported as medians with IQRs and associated *P* values. A sensitivity analysis was performed using a narrower follow-up window by excluding participants with follow-up HbA_1c_ measurements obtained within 6 weeks after study inclusion. Associations between total playtime and changes in clinical outcomes were explored using Pearson correlation coefficients. A 2-sided *P* value of <.05 was considered to indicate a possible association in these exploratory analyses.

All statistical analyses were performed using SPSS software (version 27; IBM Corp).

## Results

### Demographics

**Figure 2.**
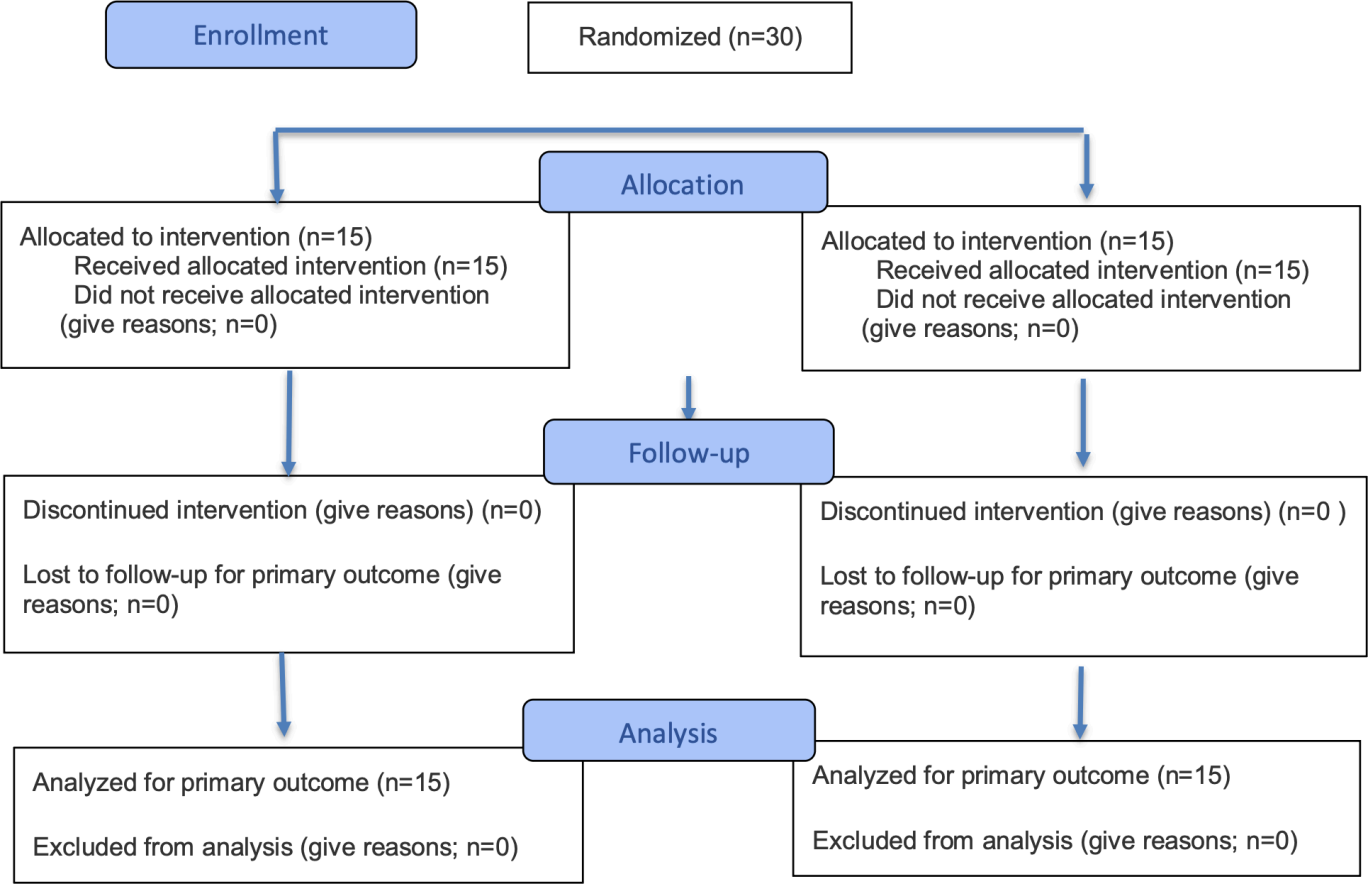
CONSORT (Consolidated Standards of Reporting Trials) 2025 flow diagram. The number of individuals assessed for eligibility prior to enrollment was not systematically recorded.

A total of 30 participants were included, of whom 16 (53.3%) were women. The mean age was 61.4 (SD 9.0) years, and the mean BMI was 32.7 (SD 6.9) kg/m². Mean baseline HbA_1c_ was 76.3 (SD 12.6) mmol/mol, and the mean duration of diabetes was 14.8 (SD 8.9; range 3‐46) years. A total of 22 (73.3%) participants used insulin, with a mean dose of 0.60 (SD 0.47) U/kg. Most participants were treated with metformin (n=23, 76.7%), whereas fewer participants used glucagon-like peptide-1 (GLP-1) receptor agonists (n=18, 60%), sulfonylureas (n=14, 46.7%), or sodium-glucose co-transporter-2 (SGLT2) inhibitors (n=7, 23.3%). All 30 participants completed the study, and no relevant data were missing for the primary or secondary outcomes. Participant flow throughout the study is shown in (Figure 2). Baseline characteristics are presented in Table 1.

**Table 1.** Baseline participant characteristics (N=30).

	Total	Control group	Intervention group
Characteristic			
Female sex, n (%)	16 (53.3)	7 (46.7)	9 (60)
Age (years), mean (SD)	61.4 (9.0)	60.3 (10.4)	62.5 (7.5)
BMI (kg/m²), mean (SD)	32.7 (6.9)	29.6 (5.1)	35.9 (7.2)
HbA_1c^a^_ (mmol/mol), mean (SD)	76.3 (12.6)	75.4 (15.4)	77.2 (9.5)
HbA_1c_ (%), mean (SD)	9.1 (1.2)	9.0 (1.4)	9.2 (0.9)
Duration of diabetes (years), mean (SD)	14.8 (8.9)	12.4 (7.0)	17.3 (10.0)
Medication			
Insulin use, n (%)	22 (73.3)	11 (73.3)	11 (73.3)
Insulin dose (U/kg), mean (SD)^b^	0.60 (0.47)	0.67 (0.59)	0.52 (0.33)
Metformin use, n (%)	23 (76.7)	9 (60)	14 (93.3)
Sulfonylurea use, n (%)	14 (46.7)	9 (60)	5 (33.3)
GLP-1^c^ receptor agonist use, n (%)	18 (60)	7 (46.7)	11 (73.3)
SGLT2^d^ inhibitor use, n (%)	7 (23.3)	2 (13.3)	5 (33.3)

a HbA_1c_: hemoglobin A_1c_.

b Insulin dose was calculated only for participants receiving insulin therapy.

c GLP-1: glucagon-like peptide-1.

d SGLT2: sodium-glucose co-transporter-2.

At baseline, participants in the intervention group had a higher BMI (mean 35.9, SD 7.2 vs mean 29.6, SD 5.1 kg/m²) and a longer duration of diabetes (mean 17.3, SD 10.0 vs mean 12.4, SD 7.0 years) compared to the control group. A greater proportion of participants in the intervention group were using metformin (n=14, 93.3% vs n=9, 60%), GLP-1 receptor agonists (n=11, 73.3% vs n=7, 46.7%), and SGLT2 inhibitors (n=5, 33.3% vs n=2, 13.3%). Medication changes occurring shortly before or during the study period were observed in 46.7% (n=7) of the participants in the intervention group and 53.3% (n=8) of the participants in the control group. Both treatment intensifications and deintensifications occurred in each group without a clear imbalance between study arms.

### Primary Outcomes

Diabetes knowledge scores increased in the intervention group from 30.0 to 36.5 but not in the control group (31.0 to 31.0). The within-group change in the intervention group was not statistically significant (*P*=.07), nor was the between-group difference (*P*=.25). Self-confidence improved from 84.0 to 92.5 in the intervention group and from 90.0 to 96.0 in the control group, while self-management increased from 62.5 to 69.5 and from 69.0 to 76.0, respectively. None of the within-group or between-group comparisons for the primary outcomes reached statistical significance after Bonferroni correction (significance threshold *P*<.017; Table 2).

**Table 2.** Outcomes before and after the intervention.

Outcome	Control group				Intervention group				Between-group, *P* value
	Pre, median (IQR)	Post, median (IQR)	Change, median (IQR)	Within-group, *P* value	Pre, median (IQR)	Post, median (IQR)	Change, median (IQR)	Within-group, *P* value	
Diabetes knowledge	31.0 (26.0 to 33.5)	31.0 (24.5 to 36.5)	2.0 (−2.0 to 4.0)	.97	30.0 (25.8 to 36.2)	36.5 (33.0 to 38.5)	3.0 (0.5 to 10.5)	.07	.25
Self-confidence (VDZ^a^)	90.0 (84.5 to 94.5)	96.0 (85.5 to 98.0)	3.0 (−0.5 to 5.5)	.08	84.0 (80.5 to 90.5)	92.5 (82.0 to 97.2)	6.0 (−0.5 to 8.5)	.07	.26
Self-management (DSMES^b^)	69.0 (62.0 to 76.5)	76.0 (69.5 to 83.5)	5.0 (1.0 to 9.5)	.02	62.5 (56.8 to 80.5)	69.5 (63.0 to 79.0)	5.0 (1.0 to 11.0)	.07	.77
HbA_1c^c^_ (mmol/mol)	70.0 (66.5 to 83.5)	68.0 (63.5 to 73.5)	−5.0 (−12.5 to 2.5)	.08	73.0 (70.8 to 81.5)	64.5 (60.8 to 72.0)	−13.0 (−17.0 to −6.5)	.007	.25
HbA_1c_ (%)	8.6 (8.2 to 9.8)	8.4 (8.0 to 8.9)	−0.5 (−1.1 to 0.2)	.08	8.8 (8.7 to 9.6)	8.0 (7.7 to 8.8)	−1.2 (−1.6 to −0.6)	.007	.25

a VDZ: Vertrouwen in Diabetes Zelfzorg.

b DSMES: Diabetes Self-Management Education and Support.

c HbA_1c_: hemoglobin A_1c_.

### Secondary Outcomes

No statistically significant between-group difference in HbA_1c_ change was observed (*P*=.25). Within-group analyses showed a significant HbA_1c_ reduction in the intervention group (73.0 to 64.5 mmol/mol; *P*=.007), whereas the smaller reduction in the control group was not statistically significant (70.0 to 68.0 mmol/mol; *P*=.08; Table 2). Baseline HbA_1c_ measurements were obtained a median of 31 (IQR 6-84) days before study inclusion, whereas follow-up HbA_1c_ measurements were obtained a median of 52 (IQR 10-82) days after inclusion. Sensitivity analysis using a narrower follow-up window yielded similar results, with median HbA_1c_ in the intervention group changing from 74.0 (IQR 70.8-85.5) to 64.5 (IQR 58.3-76.5) mmol/mol (*P*=.003).

Data are presented as median (IQR). Change scores represent postintervention minus preintervention values. *P* values represent within-group changes (Wilcoxon signed-rank test) and between-group comparisons of change scores (Mann-Whitney *U* test). The Bonferroni-corrected significance threshold for the primary outcomes was *P*<.0167.

### Playtime

Among participants in the intervention group, the mean total playtime was 524 (SD 556) minutes over 8 weeks, corresponding to just over 1 hour of gameplay per week. Participants completed 387 game sessions during the intervention period. A total of 1081 diabetes-related knowledge questions were presented, of which 894 (82.7%) were answered correctly. A moderate negative correlation was found between longer playtime and greater HbA_1c_ reduction (Pearson *r*=−0.57; *P*=.07), suggesting a possible dose-response relationship; however, this association was not statistically significant. In contrast, only a weak and nonsignificant correlation was observed between playtime and improvement in self-management scores (Pearson *r*=0.22; *P*=.51; Table 3).

**Table 3.** SugarVita gameplay and participant evaluation^a^.

Variable	Mean (SD; range)
Total minutes played	524.27 (555.57; 16.0-1734.0)
Total gameplay sessions	39.14 (48.39; 1.0-193.0)
Questions	
How enjoyable was it to play the game?	6.62 (2.18; 3.0-10.0)
How challenging was the game?	6.0 (2.35; 2.0-10.0)
How user-friendly was the game?	7.29 (1.9; 3.0-10.0)
Did you learn from the game?	8.14 (0.95; 7.0-10.0)
Would you recommend the game to others?	7.54 (2.11; 4.0-10.0)
Would you play the game more frequently?	5.25 (3.22; 1.0-10.0)
Do you think SugarVita contributes to diabetes-related knowledge?	8.04 (1.39; 5.0-10.0)
Do you think digital games are a useful support tool for people newly diagnosed with diabetes?	9.0 (1.0; 7.0-10.0)

a Descriptive statistics of gameplay activity and participant evaluation of the SugarVita serious game (intervention group only). Scores for evaluation items range from 1 (strongly disagree) to 10 (strongly agree).

### Participant Evaluation of the Intervention

All 15 participants in the intervention group completed the evaluation questionnaire, and no item responses were missing. Overall evaluations of SugarVita were favorable across several domains, using a scale from 1 (strongly disagree) to 10 (strongly agree). Agreement with the statement that SugarVita may be a useful support tool for individuals newly diagnosed with diabetes received the highest score, with a mean of 9.0 (SD 1.0) out of 10. Perceived educational value also received high ratings, with a mean of 8.1 (SD 0.9) out of 10. Most of the participants expressed willingness to recommend the game to others (mean 7.5, SD 2.1) and rated it as user-friendly (mean 7.3, SD 1.9). Ratings for enjoyment and challenge were moderate, with mean scores of 6.6 (SD 2.2) and 6.0 (SD 2.3), respectively. Willingness to continue playing the game received a lower mean score of 5.25 (SD 3.22), suggesting room for improvement in long-term engagement (Table 3).

## Discussion

### Principal Findings

In this randomized controlled pilot study, no statistically significant between-group differences were observed for the primary outcomes after correction for multiple testing. Participants in the intervention group demonstrated a significant within-group reduction in HbA_1c_, numerically higher diabetes knowledge scores, and positive evaluations of the game. However, given the absence of statistically significant between-group differences, these findings should be interpreted cautiously and cannot be attributed exclusively to the intervention. The observed changes likely reflect a combination of factors, including the intervention and routine diabetes care received during follow-up.

Serious games for diabetes self-management have been in development for several decades, with early examples targeting children with type 1 diabetes [17]. Over time, these games have evolved across various platforms, with mobile-based apps gaining popularity in recent years due to the widespread adoption of smartphones and mobile gaming. An increasing number of systematic reviews have examined the effects of diabetes-related games; however, findings remain inconclusive [9,18-20]. While most interventions have not demonstrated significant clinical effects, their potential educational value, both for patients and the research community, is increasingly acknowledged.

In this study, SugarVita was evaluated in adults with T2DM using a randomized controlled design. Over the 8-week intervention period, participants in the intervention group showed a significant within-group reduction in HbA_1c_ and numerically higher diabetes knowledge scores. While changes in self-confidence and self-management were not statistically significant, outcome measures generally changed in a favorable direction. However, no statistically significant between-group differences were observed after correction for multiple primary outcomes. The relatively small sample size and short intervention duration may have limited the ability to detect statistically significant between-group effects. Participants in both study arms received regular follow-up and structured lifestyle counseling in accordance with national guidelines. SugarVita was therefore evaluated as a complementary educational intervention alongside standard diabetes care, which may partly explain the limited between-group differences observed. In health care systems where access to diabetes education is more limited, follow-up is less frequent, or structured self-management support is less readily available, digital educational tools may yield larger relative effects.

Given these factors, the findings suggest that SugarVita may have contributed to favorable within-group changes in the setting of our study, where participants received instructions and guidance from the research team. Notably, participants rated the game as both enjoyable and educational, which may have contributed to the favorable trends observed in several outcomes. On average, they played for just over 1 hour per week. Higher total playtime was moderately associated with greater HbA_1c_ reduction, whereas the association with self-management outcomes was weak. Longer or sustained engagement with the game, such as over a longer intervention period, may be required to better evaluate its potential impact. Although these associations were not statistically significant, they may reflect a possible association between engagement and study outcomes.

The findings of this study are consistent with previous research supporting the potential of serious games to enhance diabetes self-management. Early work in children with type 1 diabetes showed that educational games could improve disease-related knowledge and behavior [17]. Among adults with T2DM, game-based interventions have demonstrated varying effects: an autonomous exercise game by Kempf and Martin [18] improved glycemic control and quality of life, and digital health games by Höchsmann et al [19] have shown promising results in promoting physical activity and adherence. Notably, a team-based online game led to significant improvements in HbA_1c_ among US veterans with T2DM [20]. In comparison with previous studies, SugarVita offered a simple, mobile-based game experience that included short educational prompts throughout gameplay. This combination of accessibility, repetition, and engagement may have contributed to the favorable trends observed in diabetes knowledge scores and HbA_1c_ levels.

Several factors may help explain the observed findings associated with SugarVita. The game was co-developed with patients with T2DM, and their preferences, including the types of games they enjoyed, were carefully considered during development. This likely contributed to the game’s relevance and acceptability. SugarVita is designed to be easily accessible and simple to integrate into daily life. Players engage in everyday scenarios that reflect real-life situations, offering a sense of recognition and familiarity. Importantly, players receive immediate feedback on how common daily choices influence blood glucose levels, which may enhance learning through direct cause-and-effect visualization. Educational prompts are short and integrated into gameplay, supporting incidental learning—especially for those who may not benefit as much from traditional educational formats such as one-on-one consultations with health care providers, which can sometimes feel abstract or fail to help patients visualize the consequences of their actions. Participant evaluations confirmed that the game was perceived as educational, user-friendly, and especially useful for newly diagnosed patients with diabetes. However, ratings for playability and challenge were moderate, and willingness to continue playing was relatively low, with variation between participants, suggesting that long-term engagement could be improved. Future iterations of SugarVita could address this by introducing more progressive challenges and greater variation in difficulty over time. In addition, expanding social and multiplayer features, such as collaborative or competitive challenges, and improving personalization and visual feedback may help sustain player interest, ensure the game remains both valuable and challenging for returning players, and support longer-term intervention effects. Player performance within the game was not included in the analyses, as comparing in-game scores was complicated by the use of weekly assignments and variation in game modes; therefore, total playtime was used as the main indicator of engagement.

This study has several strengths. It was conducted as a randomized controlled trial, applying a structured design to evaluate the impact of a digital intervention in people with T2DM. Validated instruments were used to assess self-management and self-confidence, while diabetes-related knowledge was assessed using a translated version of the Diabetes Knowledge Test that had not yet been formally validated in Dutch. Real-world HbA_1c_ values were obtained from routine care without requiring additional blood draws, minimizing participant burden. The game was co-designed with the target population, increasing its practical relevance and user engagement. In addition, player data, such as frequency and duration of gameplay, were objectively monitored, enabling the exploration of potential dose-response relationships.

Several limitations should also be noted. The sample size was modest, limiting statistical power, particularly for secondary outcomes. This study was designed as a randomized controlled trial with a parallel-group design; however, as an exploratory pilot study, no formal power calculation was performed. Randomization was stratified based on age and baseline HbA_1c_; however, some baseline differences remained between groups, particularly in BMI, diabetes duration, and glucose-lowering medication use. Although these differences were not statistically significant, they may have influenced the observed outcomes, especially HbA_1c_. Baseline use of GLP-1 receptor agonists was more common in the intervention group. Review of medication changes showed both treatment intensifications and discontinuations of GLP-1 receptor agonists within the intervention group, while initiation and dose escalation also occurred in the control group. Nevertheless, the potential influence of baseline differences in glucose-lowering medication use and medication changes during follow-up on glycemic outcomes cannot be excluded. In addition, participants were intentionally recruited on the basis of elevated HbA_1c_ levels (>64 mmol/mol), which may have increased the likelihood of regression to the mean. Therefore, the within-group HbA_1c_ reduction observed in the intervention group should be interpreted cautiously and cannot be attributed solely to the SugarVita intervention.

The diabetes knowledge questionnaire, although based on a widely used instrument, had not been formally validated in Dutch at the time of the study. Consequently, findings related to diabetes knowledge should be interpreted with caution and regarded as exploratory. Future studies should preferably use a formally validated Dutch instrument or validate the translated version prior to implementation. In addition, repeated administration of the same questionnaires may have introduced practice effects, particularly for the diabetes knowledge assessment.

Participant evaluations may have been influenced by social desirability bias and the Hawthorne effect, potentially contributing to more favorable ratings. Furthermore, digital literacy, access to digital devices, and prior experience with mobile apps or games were not formally assessed. These factors may have influenced engagement with the intervention and its perceived usability.

Furthermore, follow-up was limited to the short-term effects directly following the intervention, so the durability of these benefits remains unknown. In addition, the game was tested in a single center, which may limit the generalizability of the findings to broader or more diverse populations. Finally, the relatively high level of baseline diabetes care in this setting may limit the generalizability of the findings to health care systems with different levels of access to diabetes education.

### Conclusions

SugarVita, a mobile serious game co-developed with patients, was perceived as educational and user-friendly by participants. No statistically significant between-group differences were observed for the primary and secondary outcomes. These findings support the feasibility and acceptability of SugarVita as a digital educational intervention for people with T2DM.

Given the global rise in T2DM and the growing need for scalable educational interventions, serious games such as SugarVita may represent a valuable complementary approach to diabetes education and self-management. Accessibility was a key consideration in the game’s development. Future work should focus on expanding in-game options and tailoring content to sustain engagement and increase relevance across diverse patient populations. Larger, adequately powered studies with longer follow-up are needed to determine effectiveness and optimize sustained engagement.

## Supplementary material

10.2196/99345 Multimedia Appendix 1 Assignment card used during the SugarVita intervention.

10.2196/99345 Multimedia Appendix 2 Participant evaluation form completed after the intervention.

10.2196/99345 Multimedia Appendix 3 Questionnaires used to assess diabetes knowledge, self-efficacy, and self-management.

10.2196/99345 Checklist 1 CONSORT checklist.

## Abbreviations

CONSORT: Consolidated Standards of Reporting Trials
GLP-1: glucagon-like peptide-1
HbA_1c_: hemoglobin A_1c_
SGLT2: sodium-glucose co-transporter-2
T2DM: type 2 diabetes mellitus
