# Supplementary material for: A Serious Digital Game (SugarVita) to Support Diabetes Self-Management: Pilot Randomized Controlled Trial
Source: JMIR Diabetes. 2026 Aug 7;11:e99345. doi: 10.2196/99345 (PMC13451009; doi:10.2196/99345)
Supplement: Supplementary file 1 [file diabetes-v11-e99345-s001.pdf]

máxima mc

SUGARVITA

TU/e EINDHOVEN UNIVERSITY OF TECHNOLOGY

Opdrachtenkaart SugarVita

Week 1: kennismaking, in de eerste week kunt u kennismaken met SugarVita, er zijn geen specifieke opdrachten

Week 2: educatie, probeer 10 vragen op rij goed te beantwoorden en speel daarmee een nieuwe pion vrij

Week 3: voeding, speel een rondje waarbij je kiest voor koolhydraat-rijke en vervolgens koolhydraat-arme voeding

Week 4: samen spelen, speel een rondje samen met je partner, vrienden of familie en leer ze iets nieuws over diabetes

Week 5: medicatie, speel een rondje waar je je medicatie niet mag gebruiken

Week 6-8: vrij spel, probeer een extra pion vrij te spelen door in totaal 25 vragen goed te hebben beantwoord

Uw vragen en notities:
