# Supplementary material for: A Serious Digital Game (SugarVita) to Support Diabetes Self-Management: Pilot Randomized Controlled Trial
Source: JMIR Diabetes. 2026 Aug 7;11:e99345. doi: 10.2196/99345 (PMC13451009; doi:10.2196/99345)
Supplement: Supplementary file 2 [file diabetes-v11-e99345-s002.pdf]

Studienummer: ………..

Datum………………

Evaluatieformulier SugarVita

U bent gevraagd om het diabetesspel SugarVita afgelopen periode tijdens het onderzoek te spelen. Graag willen wij hier uw mening over weten, zodat wij het spel nog verder kunnen verbeteren.

Welk cijfer geeft u het spel op de volgende punten, waarbij 1 staat voor 'helemaal niet' en 10 voor 'heel erg'? (omcirkel het getal). Bij uitleg kunt u uw cijfer onderbouwen.

Hoe leuk is het spel om te spelen?

1 2 3 3 5 6 7 8 9 10

Uitleg:

Hoe uitdagend is het spel om te spelen?

1 2 3 4 5 6 7 8 9 10

Uitleg:

Hoe gebruiksvriendelijk is het spel?

1 2 3 4 5 6 7 8 9 10

Uitleg:

Kunt u van het spel leren?

1 2 3 4 5 6 7 8 9 10

Uitleg:

Zou u het spel aan anderen aanraden?

1 2 3 4 5 6 7 8 9 10

Uitleg:

Studienummer: ………..   Datum………………

Zou u het spel vaker spelen?

1 2 3 4 5 6 7 8 9 10

Uitleg:

Denkt u dat SugarVita, een zinvolle bijdrage kan leveren om uw kennis over diabetes te vergroten?

1 2 3 4 5 6 7 8 9 10

Uitleg:

Denkt u dat digitale spellen, zoals SugarVita, een goede ondersteuning kunnen zijn voor mensen die recent te horen hebben gekregen dat zij diabetes hebben?

1 2 3 4 5 6 7 8 9 10

Uitleg:

Wat vond u het leukst aan SugarVita?

Uitleg:

Wat vond u het minst leuk aan SugarVita?

Uitleg:

Heeft u verbeterpunten voor SugarVita?

Uitleg:

Einde evaluatieformulier. Hartelijk bedankt voor uw medewerking.
