# Supplementary material for: A Serious Digital Game (SugarVita) to Support Diabetes Self-Management: Pilot Randomized Controlled Trial
Source: JMIR Diabetes. 2026 Aug 7;11:e99345. doi: 10.2196/99345 (PMC13451009; doi:10.2196/99345)
Supplement: Supplementary file 3 [file diabetes-v11-e99345-s003.pdf]

Studienummer: ……….. **Datum………………**

Vragenlijst start studie

Deel 1: Algemene diabetes kennis vragen

Kies één antwoord welke het meest van toepassing is; tenzij anders is aangegeven.

1. Welke bloedsuikerwaarde is goed? Kies één van de onderstaande opties:
0 tussen 4,5 en 8 mmol/L
0 lager dan 5 mmol/L
0 tussen 5 en 10 mmol/L
0 tussen 3,5 en 15 mmol/L
0 bij waarden waarbij ik geen klachten heb
0 weet ik niet

2. Hoe kunt u structureel te hoge bloedsuiker verbeteren? Kruis aan wat van toepassing is; meerdere antwoorden kunnen worden ingevuld.
0 geen actie
0 dagelijks een half uur wandelen
0 extra water drinken
0 meer diabetesmedicatie
0 extra eten
0 afname van gewicht
0 weet ik niet

3. Waardoor kan de bloedsuiker stijgen? Kruis aan wat van toepassing is; meerdere antwoorden kunnen worden ingevuld.
0 u heeft te veel diabetes medicatie ingenomen
0 indien u grieperig bent
0 na het eten van een extra portie aardappelen
0 na het eten van een extra portie vlees
0 na het eten van een mandarijn
0 bij gewichtstoename
0 weet ik niet

4. Bij welk van de onderstaande producten zal uw bloedsuiker het meeste stijgen? Kies één van de onderstaande opties:
0 knäckebröd met jam
0 zacht broodje met kaas
0 één krentenbol
0 één snee bruin brood met kaas
0 één snee wit brood met kaas
0 weet ik niet

Studienummer: ……….. Datum………………

5. U bent thuis en voelt zich trillerig, wat zweterig en heeft honger. U heeft geen druivensuikers bij de hand. Wat zou u doen? Kies één van de onderstaande opties:

0 u wacht even af
0 een wandeling maken
0 een boekje lezen
0 een chocoladereep eten
0 glas gewone frisdrank
0 een boterham met kaas eten
0 weet ik niet

6. Wanneer verwacht u dat uw bloedsuiker zal dalen? Kruis aan wat van toepassing is; meerdere antwoorden kunnen worden ingevuld.

0 huishoudelijk werk (stofzuigen, ramen wassen)
0 tv kijken
0 afwassen
0 tuinieren
0 na een middag winkelen
0 veel water drinken
0 weet ik niet

7. Bij welk van de onderstaande producten zal uw bloedsuiker het meeste stijgen? Kruis aan wat van toepassing is; meerdere antwoorden kunnen worden ingevuld.

0 schaaltje rauwkost
0 vruchten smoothie
0 zakje chips
0 jus d'orange
0 kom heldere groentesoep
0 blokjes kaas
0 weet ik niet

8. U gaat een uurtje fietsen. Wat verwacht u dat er met de bloedsuiker gaat gebeuren? Kies één van de onderstaande opties:

0 de bloedsuiker stijgt
0 de bloedsuiker blijft gelijk
0 de bloedsuiker daalt
0 ik weet het niet

9. Welk ontbijt bevat de meeste koolhydraten (suikers)? Kies één van de onderstaande opties:
0 1 snee brood met zoet beleg
0 1 snee brood met hartig beleg
0 1 schaaltje yoghurt met 3 lepels cruesli
0 1 cracker met zoet beleg
0 weet ik niet

10.Welke van onderstaande dranken hebben <u>geen</u> invloed op uw bloedsuiker? Kruis aan wat van toepassing is; meerdere antwoorden kunnen worden ingevuld.
0 koffie of thee met zoetje
0 koffie of thee met 1 klontje suiker
0 dubbelfris light
0 cola light
0 verse jus d'orange sap
0 slimpie aanmaaklimonade
0 roosvicee
0 weet ik niet

11. Welke van de onderstaande beweringen zijn waar?
A: 1 portie vanille vla bevat evenveel suikers als 1 portie yoghurt
B: 3 middelgrote aardappelen bevat evenveel suikers als 3 sneetjes brood
0 A en B zijn waar
0 A is waar
0 B is waar
0 A en B zijn niet waar
0 weet ik niet

12. Hoe ziet u op een etiket hoeveel suiker een product bevat:
0 aan de koolhydraten
0 aan de regel: "waarvan suikers"
0 aan de energie/ kilocalorieën
0 aan de eiwitten
0 weet ik niet

13. Het geadviseerde dieet voor mensen met diabetes is:
0 De manier waarop de meeste Nederlanders eten
0 Een gezond dieet voor de meeste mensen
0 Te veel koolhydraten voor de meeste mensen
0 Te veel eiwitten voor de meeste mensen

14. Welk effect heeft ongezoet vruchtensap op bloedsuikerspiegel?
0 Verlaagt het
0 Verhoogt het
0 Heeft geen effect

Studienummer: ……….. **Datum………………**

15. U heeft een hypo, met flink transpireren , hongergevoel en trillen en gaat hiervoor druivensuiker nemen. Hoeveel tabletten heeft u hiervoor nodig

0 1 tablet
0 2 tabletten
0 3 tabletten
0 5-7 tabletten

Onderstaande vragen hoeven alleen te worden beantwoord als u zelf insuline gebruikt. Indien u geen insuline gebruikt, hoeven deze vragen niet te worden beantwoord.

----

16. Als je de griep hebt, is het verstandig om:
0 Minder insuline te spuiten
0 Minder te drinken
0 Meer eiwitten te eten
0 De bloedsuikerspiegel vaker te meten

17. Als je in de ochtend kortwerkende insuline hebt gespoten maar het ontbijt overslaat, zal je bloedsuikerspiegel meestal:
0 Stijgen
0 Dalen
0 Hetzelfde blijven

18. Als je kortwerkende insuline hebt gespoten, is de kans op een lage bloedsuikerspiegel het grootst binnen:
0 Minder dan 2 uur
0 3-5 uur
0 6-12 uur
0 Meer dan 13 uur

19. Vlak voor de lunch ontdekt je dat je de insuline bij het ontbijt bent vergeten te spuiten. Wat moet je nu doen?
0 Je lunch overslaan om je bloedsuikerspiegel te verlagen.
0 Dezelfde dosering insuline spuiten die je normaal bij het ontbijt spuit
0 Tweemaal zoveel insuline spuiten die je normaal bij het ontbijt spuit
0 Je bloedsuikerspiegel meten om te beslissen hoeveel insuline je moet spuiten

------------------------- Einde vragenlijst 1---

Studienummer: ……….. **Datum………………**

Deel 2: Diabetes Management (DMSES)

Toelichting:
Wij willen graag van u weten of u denkt (= uw eigen inschatting) dat u in staat bent de verschillende handelingen uit te voeren die nodig zijn om met uw diabetes om te gaan.
Bij de 20 vragen kunt u kiezen uit 5 antwoorden. Er is maar één antwoord mogelijk. Kies het antwoord dat het beste uw mening weergeeft (X).

1. Ik denk dat ik in staat ben om mijn bloedsuiker zelf te controleren als dat nodig is:
( ) waarschijnlijk niet
( ) misschien wel, misschien niet
( ) waarschijnlijk wel
( ) hoogstwaarschijnlijk wel
( ) zeker wel

2. Ik denk dat ik in staat ben om een te hoge bloedsuiker te verhelpen:
( ) waarschijnlijk niet
( ) misschien wel, misschien niet
( ) waarschijnlijk wel
( ) hoogstwaarschijnlijk wel
( ) zeker wel

3. Ik denk dat ik in staat ben om een te lage bloedsuiker te verhelpen:
( ) waarschijnlijk niet
( ) misschien wel, misschien niet
( ) waarschijnlijk wel
( ) hoogstwaarschijnlijk wel
( ) zeker wel

4. Ik denk dat ik in staat ben om zelf de juiste voeding voor mijn diabetes te kiezen:
( ) waarschijnlijk niet
( ) misschien wel, misschien niet
( ) waarschijnlijk wel
( ) hoogstwaarschijnlijk wel
( ) zeker wel

5. Ik denk dat ik in staat ben om zelf in mijn voeding variatie aan te brengen:
( ) waarschijnlijk niet
( ) misschien wel, misschien niet
( ) waarschijnlijk wel
( ) hoogstwaarschijnlijk wel
( ) zeker wel

Studienummer: ……….. **Datum………………**

6. Ik denk dat ik in staat ben om zelf mijn gewicht onder controle te houden:
( ) waarschijnlijk niet
( ) misschien wel, misschien niet
( ) waarschijnlijk wel
( ) hoogstwaarschijnlijk wel
( ) zeker wel

7. Ik denk dat ik in staat ben om zelf mijn voeten op wondjes te controleren:
( ) waarschijnlijk niet
( ) misschien wel, misschien niet
( ) waarschijnlijk wel
( ) hoogstwaarschijnlijk wel
( ) zeker wel

8. Ik denk dat ik in staat ben om zelf te zorgen voor voldoende lichaamsbeweging, door bijvoorbeeld te wandelen of te fietsen:
( ) waarschijnlijk niet
( ) misschien wel, misschien niet
( ) waarschijnlijk wel
( ) hoogstwaarschijnlijk wel
( ) zeker wel

9. Ik denk dat ik in staat ben om mijn voeding aan te passen, wanneer ik ziek ben:
( ) waarschijnlijk niet
( ) misschien wel, misschien niet
( ) waarschijnlijk wel
( ) hoogstwaarschijnlijk wel
( ) zeker wel

10. Ik denk dat ik in staat ben om mij meestal aan mijn voedingsvoorschriften te houden:
( ) waarschijnlijk niet
( ) misschien wel, misschien niet
( ) waarschijnlijk wel
( ) hoogstwaarschijnlijk wel
( ) zeker wel

11. Ik denk dat ik in staat ben om aan extra lichaamsbeweging te doen, wanneer dit door de arts wordt geadviseerd:
( ) waarschijnlijk niet
( ) misschien wel, misschien niet
( ) waarschijnlijk wel
( ) hoogstwaarschijnlijk wel
( ) zeker wel

12. Ik denk dat ik in staat ben om mijn voeding, als dit nodig is, aan te passen, wanneer ik extra lichaamsbeweging neem:
( ) waarschijnlijk niet
( ) misschien wel, misschien niet
( ) waarschijnlijk wel
( ) hoogstwaarschijnlijk wel
( ) zeker wel

13. Ik denk dat ik in staat ben om mij aan mijn voedingsvoorschriften te houden, wanneer ik van huis ben:
( ) waarschijnlijk niet
( ) misschien wel, misschien niet
( ) waarschijnlijk wel
( ) hoogstwaarschijnlijk wel
( ) zeker wel

14. Ik denk dat ik in staat ben om mijn voeding aan te passen, wanneer ik van huis ben:
( ) waarschijnlijk niet
( ) misschien wel, misschien niet
( ) waarschijnlijk wel
( ) hoogstwaarschijnlijk wel
( ) zeker wel

15. Ik denk dat ik in staat ben om mij aan mijn voedingsvoorschriften te houden, wanneer ik op vakantie ben:
( ) waarschijnlijk niet
( ) misschien wel, misschien niet
( ) waarschijnlijk wel
( ) hoogstwaarschijnlijk wel
( ) zeker wel

16. Ik denk dat ik in staat ben om mij aan mijn voedingsvoorschriften te houden, wanneer ik op een receptie of feestje ben:
( ) waarschijnlijk niet
( ) misschien wel, misschien niet
( ) waarschijnlijk wel
( ) hoogstwaarschijnlijk wel
( ) zeker wel

Studienummer: ……….. Datum………………

17. Ik denk dat ik in staat ben om bij spanning of stress mijn voeding aan te passen:
( ) waarschijnlijk niet
( ) misschien wel, misschien niet
( ) waarschijnlijk wel
( ) hoogstwaarschijnlijk wel
( ) zeker wel

18. Ik denk dat ik in staat ben om één keer per jaar de dokter te raadplegen voor controle van mijn diabetes:
( ) waarschijnlijk niet
( ) misschien wel, misschien niet
( ) waarschijnlijk wel
( ) hoogstwaarschijnlijk wel
( ) zeker wel

Wilt u de volgende twee vragen nog invullen wanneer u tabletten voor uw diabetes gebruikt. Wanneer u geen tabletten voor uw diabetes gebruikt kunt u de vragen 19 en 20 overslaan

----

19. Ik denk dat ik in staat ben om mijn tabletten volgens voorschrift in te nemen, wanneer ik van huis ben:
( ) waarschijnlijk niet
( ) misschien wel, misschien niet
( ) waarschijnlijk wel
( ) hoogstwaarschijnlijk wel
( ) zeker wel

20. Ik denk dat ik in staat ben om mijn dosering tabletten aan te passen, wanneer ik ziek ben:
( ) waarschijnlijk niet
( ) misschien wel, misschien niet
( ) waarschijnlijk wel
( ) hoogstwaarschijnlijk wel
( ) zeker wel

------------------------- Einde vragenlijst 2--

Studienummer: ……….. **Datum………………**

Deel 3: Vertrouwen in diabetes zelfzorg (VDZ)

Toelichting: In de behandeling van diabetes heeft u zelf als patiënt een belangrijk aandeel. Hieronder wordt u gevraagd om aan te geven hoeveel vertrouwen u erin heeft dat u de verschillende zelfzorgactiviteiten kunt uitvoeren. Daartoe **omcirkelt** u bij onderstaande uitspraken het getal dat het beste weergeeft in welke mate u denkt dat u in staat bent de genoemde activiteit uit te voeren. Het gaat er dus niet om of u dit daadwerkelijk doet, maar in hoeverre u er op dit moment vertrouwen in heeft dat u het kunt.

**Ik denk dat ik in staat ben om ...**	nee, dat kan ik zeker niet	nee, ik geloof niet dat ik dat kan	ik weet niet of ik dat kan	ja, ik geloof dat ik dat kan	ja, dat kan ik zeker
**1**. ... alle activiteiten uit te voeren die nodig zijn om mijn diabetes goed te reguleren.	1	2	3	4	5
**2**. ... bij maaltijden en tussendoortjes rekening te houden met de voedingsadviezen.	1	2	3	4	5
**3.** ... mijn bloedglucose tenminste twee keer per dag te controleren.	1	2	3	4	5
**4.** ... mijzelf elke dag het voorgeschreven aantal insuline-injecties toe te dienen.	1	2	3	4	5
**5.** ... mijn insuline aan te passen in bijzondere situaties (sport, reizen, feestjes).	1	2	3	4	5
**6.** ... mijn insuline aan te passen wanneer ik ziek ben.	1	2	3	4	5
**7**. ... een hoge bloedglucose vroeg genoeg op te merken om te kunnen corrigeren.	1	2	3	4	5
**8**. ... een lage bloedglucose vroeg genoeg op te merken om te kunnen corrigeren.	1	2	3	4	5
**9.** ... een hoge bloedglucose op de juiste manier te corrigeren.	1	2	3	4	5
**10**. ... een lage bloedglucose op de juiste manier te corrigeren.	1	2	3	4	5
**11.** ... mijn bloedglucose-waarden dagelijks bij te houden in een dagboekje.	1	2	3	4	5
**12**. ... te beslissen wanneer het nodig is om een arts of verpleegkundige te raadplegen.	1	2	3	4	5

Studienummer: ……….   **Datum………………**

**Ik denk dat ik in staat ben om ...**	nee, dat kan ik zeker niet	nee, ik geloof niet dat ik dat kan	ik weet niet of ik dat kan	ja, ik geloof dat ik dat kan	ja, dat kan ik zeker
**13**. ... mijn arts vragen te stellen over de behandeling van mijn diabetes.	1	2	3	4	5
**14**. ... bij spanningen (stress) mijn bloedglucose binnen normale grenzen te houden.	1	2	3	4	5
**15**. ... mijn voeten dagelijks te inspecteren op blaren en wondjes.	1	2	3	4	5
**16**. ... vrienden/ familie te vragen mij te helpen met mijn diabetes.	1	2	3	4	5
**17**. ... collega's/ anderen van mijn diabetes op de hoogte te brengen wanneer dat nodig is.	1	2	3	4	5
**18**. ... mij aan mijn afspraken op de polikliniek te houden.	1	2	3	4	5
**19**. ... twee tot drie maal per week aan sport/ lichaamsbeweging te doen.	1	2	3	4	5
**20**. ... een goede keuze uit het menu te maken wanneer ik uit eten ben.	1	2	3	4	5
**21**. ... over complicaties van diabetes te lezen/ te horen zonder ontmoedigd te raken.	1	2	3	4	5

------------------------- Einde vragenlijsten --
